# Cost-effectiveness analysis for imaging techniques with a focus on cardiovascular magnetic resonance

**DOI:** 10.1186/1532-429X-15-52

**Published:** 2013-06-14

**Authors:** Sanjeev A Francis, Caroline Daly, Bobak Heydari, Siddique Abbasi, Ravi V Shah, Raymond Y Kwong

**Affiliations:** 1 Department of Medicine, Brigham and Women's Hospital, Cardiovascular Division, Boston, MA, USA; 2 Cardiology Division, Department of Medicine, Massuchusetts General Hospital, Boston, MA, USA; 3 Cardiology Division, St. James’ Hospital, Dublin, Ireland

## Abstract

With the need for healthcare cost-containment, increased scrutiny will be placed on new medical therapeutic or diagnostic technologies. Several challenges exist for a new diagnostic test to demonstrate cost-effectiveness. New diagnostic tests differ from therapeutic procedures due to the fact that diagnostic tests do not generally directly affect long-term patient outcomes. Instead, the results of diagnostic tests can influence management decisions for patients and by this route, diagnostic tests indirectly affect long-term outcomes. The benefits from a specific diagnostic technology depend therefore not only on its performance characteristics, but also on other factors such as prevalence of disease, and effectiveness of existing treatments for the disease of interest. We review the concepts and theories of cost-effectiveness analyses (CEA) as they apply to diagnostic tests in general. The limitations of CEA across different study designs and geographic regions are discussed, and we also examine the strengths and weakness of the existing publications where CMR was the focus of CEA compared to other diagnostic options.

## Review

“It is now almost universally believed that the resources available to meet the demands for health care are limited. This fact was not, perhaps, perceived to be so a few decades ago, before health insurance became so pervasive and before medical technologies had proliferated to the extent that they have today” [1].

In a seminal paper in 1977, Weinstein and Stason introduced the concept of cost-effectiveness in health care to a broad clinical audience [1]. Over the ensuing decades, the health care environment (and available technologies) has continued to evolve. The limit of health care resources is even more pronounced in the current economic climate, forcing a balance between fiscal restraint and optimal use of resources to maximize health. In this setting, cardiac imaging has come under increased scrutiny, owing to increased use and unclear benefit. In this review, we discuss the framework of cost-effectiveness analysis (CEA) and its application in cardiac imaging, specifically focusing on cardiovascular magnetic resonance (CMR).

### The current climate: why CEA is necessary

**Figure 1:**
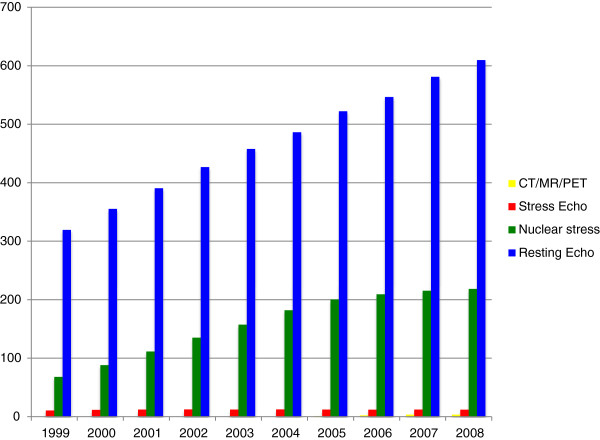
**Non-invasive imaging services provided by cardiologists per 1000 Medicare beneficiaries from 1999–2008.** CMR accounts for a very small percentage of the total expenditures for cardiac imaging amongst Medicare beneficiaries. Data adapted from Andrus et al. [5].

Cardiovascular disease remains the leading cause of death worldwide [2]. Since 1968, there has been a steady decline in deaths from coronary heart disease in the United States [3]. During this same interval, there has been an increase in health care expenditures attributable to heart disease, with an estimated cost of $316 billion dollars in 2010 secondary to health care expenditures and lost productivity [4]. An analysis of Medicare claims between 1999–2008 revealed that 78% of the growth in cardiovascular services was attributed to non-invasive testing, relative to invasive procedures and evaluation and management (E&M), which contributed 5% and 17%, respectively [5]. Despite rapid growth in advanced cardiac imaging modalities (e.g., CT/CMR/PET), these modalities accounted for a small percentage of the increased cost, relative to nuclear stress imaging and echocardiography (accounted for 48% of the total growth in services; Figure 1). A shift in imaging services from the inpatient to outpatient setting is one of the major factors associated with this rapid increase in cardiac imaging in the past decade [6].

In the past decades, very few studies have made the effort of relating the growth in imaging to direct, cost-effective improvement in patient care. Attempts to link increased use of cardiac imaging to improved downstream outcomes have also been difficult, given that assessing the contribution of diagnostic accuracy is difficult. For example, in a cross sectional population based study of Medicare patients from 1993–2001, there was a 3-fold increase in imaging stress tests, matching an increase in cardiac catheterization and revascularization rates and a near 50% decline in the age-adjusted rate of coronary heart disease mortality between 1980–2000 [7]. The authors concluded that approximately half of this reduction in cardiac mortality was attributed to modification of major risk factors and the other half attributable largely to evidence-based medical therapies for acute coronary syndromes and heart failure. In practice, while diagnostic imaging contributes in the decision-making to enact primary or secondary prevention strategies as well as the decision to undergo revascularization, the impact from the diagnostic information that influences therapy is not easily quantifiable. Given rapidly increasing costs and concerns around the efficacy of diagnostic imaging, cardiac imaging has become a target of various cost-saving measures. In 2010, the Centers for Medicare and Medicaid Services (CMS) physician fee schedule included a near 40% reduction in reimbursement for nuclear imaging and echocardiography [8]. These cuts are continued in the proposed 2013 physician fee schedule with a call for an additional 3% reduction in reimbursement driven in part by decreased payments for advanced cardiac imaging procedures [9]. This trend in increased growth in cardiac imaging and greater scrutiny on cost saving measures is not limited to the United States. The experience in Canada has been similar with growth in cardiac imaging ranging from 5-10% annually between 1992 and 2001, outpacing changes in the prevalence of coronary artery disease [10]. In Europe, the utilization pattern is different with a 44% increase in the number of CMR scans in the United Kingdom from 2008–2010 [11]. It is clear that cost-effectiveness analysis is critical for the appropriate use of cardiac imaging. This is particularly relevant for the future growth of CMR, given its costs and the increasing scrutiny from third party payers.

### Cost-effectiveness analysis: a brief primer

Cost-effectiveness analysis provides a framework upon which to compare different management strategies or treatments through the prism of maximizing health benefit within the constraint of limited resources [1]. Though often used interchangeably cost-benefit, cost-effectiveness and cost-utility are related but distinct. In a cost-benefit analysis both the cost and the benefit, which in this case is health, are measured in monetary terms. This requires placing a dollar value on health and therefore life. While this methodology is appropriate in the analysis of many economic systems, placing a direct monetary value on human health can be problematic. As a result many health economists prefer cost effectiveness which describes the relationship between cost and a measure of health relevant to the intervention being analyzed such as life-years gained, disease free survival in a cancer treatment study, or reductions in blood pressure in a trial of an anti-hypertensive.

**Figure 2:**
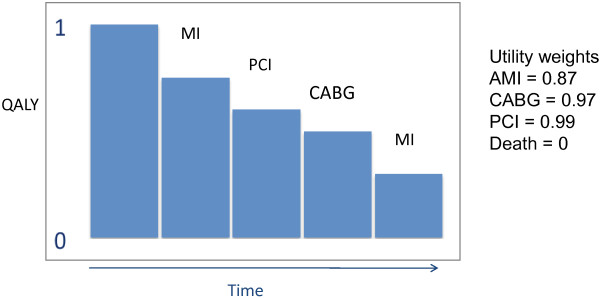
**Quality adjusted life years and utility.** By convention perfect health is assigned a value of 1 and death is assigned a value of 0. Various health states such as myocardial infarction, percutaneous coronary intervention, coronary artery bypass surgery are assigned a utility weight which reflects the estimated effect on total health. Utility weights in this example were obtained from http://www.cearegistry.org.

The central metric of cost-effectiveness is the cost-effectiveness ratio (C/E) with cost in the numerator (in dollars, Euros, etc.) and effectiveness in the denominator [12,13]. In the context of a CEA the cost of a particular therapy is the sum of all resources consumed. This includes the direct cost of care (i.e. hospital, drugs, treatments, diagnostic tests, physician fees), indirect costs (i.e. lost productivity from work, travel, day care), as well as intangible costs (i.e. pain, suffering). In the most basic sense, the “effectiveness” of a treatment can be represented as the years of life gained. However, for most interventions improvements in quality of life are more prominent, giving rise to a quality-adjusted life year (QALY). The QALY applies a weighting factor to account for varying degrees of health for each year of life gained or lost. By convention perfect health is assigned a value of 1 and death a value of 0. For all of the health states in between there is a deduction in QALY (Figure 2). A cost utility analysis is a type of cost-effective analysis that uses QALY to measure treatment effect and allows for the comparison of different treatments across different diseases such as dialysis in end stage kidney disease vs. transcatheter aortic valve replacement for aortic stenosis. Cost-utility analyses are important from a public health policy and societal standpoint. One example of a registry resource that contains independently-reviewed information on QALY, utility weighting, and other metrics relevant to CEA under various disease classification, is the Cost-Effectiveness Analysis Registry developed by the Institute for Clinical Research and Health Policy Studies of Tufts Medical Center in Boston (https://research.tufts-nemc.org/cear4).

For a particular treatment or test, the C/E ratio in isolation is of little value, unless compared to an alternative treatment/test (or no treatment/test). C (“comparative effectiveness”) ratio as detailed in the formula below:

$$\begin{array}{l} \mathrm{Incremental}\;\mathrm{Cost}\;\mathrm{Effectiveness}\;\mathrm{Ratio}\\ \;=\frac{{\mathrm{Cost}}_{\mathrm{new}\;\mathrm{strategy}}–{\mathrm{Cost}}_{\mathrm{current}\;\mathrm{practice}}}{{\mathrm{Effect}}_{\mathrm{new}\;\mathrm{strategy}}–{\mathrm{Effect}}_{\mathrm{current}\;\mathrm{practice}}} \end{array}$$

When two therapies are equivalent in their effect a cost-minimization analysis can be performed. The rationale being that if two therapies are equal the cheaper one is favorable from an economic perspective. In reality it is often very difficult to determine clinical equivalence and caution must be used when interpreting results from such analyses. When clinical equivalence is demonstrated through rigorous trial data then a cost-minimization analysis can be a useful economic tool.

There are several important aspects of CEA including perspective, time horizon, and cost-effective thresholds, which influence how results should be interpreted. Depending on the specific health care system, there are many different stakeholders: governments, insurance companies, hospitals, physicians, employers, and patients. Each participant has a different perspective with regard to cost and effect. It is important to recognize that what may be cost-effective from a societal standpoint may not be from the perspective of an individual hospital or medical practice. The societal perspective looks at the aggregate costs and effects on all members and is the one most often employed in CEA [14].

The time horizon or the period of time for which the analysis is conducted impacts the assessment of both cost and effect. Short-term vs. long-term costs/effects may be very different and thus can alter the C/E ratio of a given therapy depending on the time horizon used in the analysis. There are widely referenced thresholds which are sometimes used to define the boundaries of a cost-effective therapy. In the U.S. $50 K/QALY is used while in the UK £20 K/QALY. These thresholds may not necessarily reflect a society’s willingness to pay and some have suggested alternative methods to determine what is a cost-effective therapy using annual average income [15]. This threshold value is dependent on regional economic status and cultural factors, thus is variable between nations or even regions.

There are several misconceptions regarding CEA. Cost-effective does not necessarily mean cost-savings. The cheaper treatment is not always the most cost-effective. In the same manner, the most effective treatment/test is not always the most cost-effective. The value of a treatment or test is defined in terms of its relative benefit at an incremental cost. CEA can provide a framework for comparing different treatments/management strategies and allow health policy makers to determine whether the incremental benefit is worth the incremental cost [1,16]. For a more detailed description of the principles of cost effectiveness analysis the reader is directed to several reviews [17]–[19].

### Formulating the decision options

**Figure 3:**
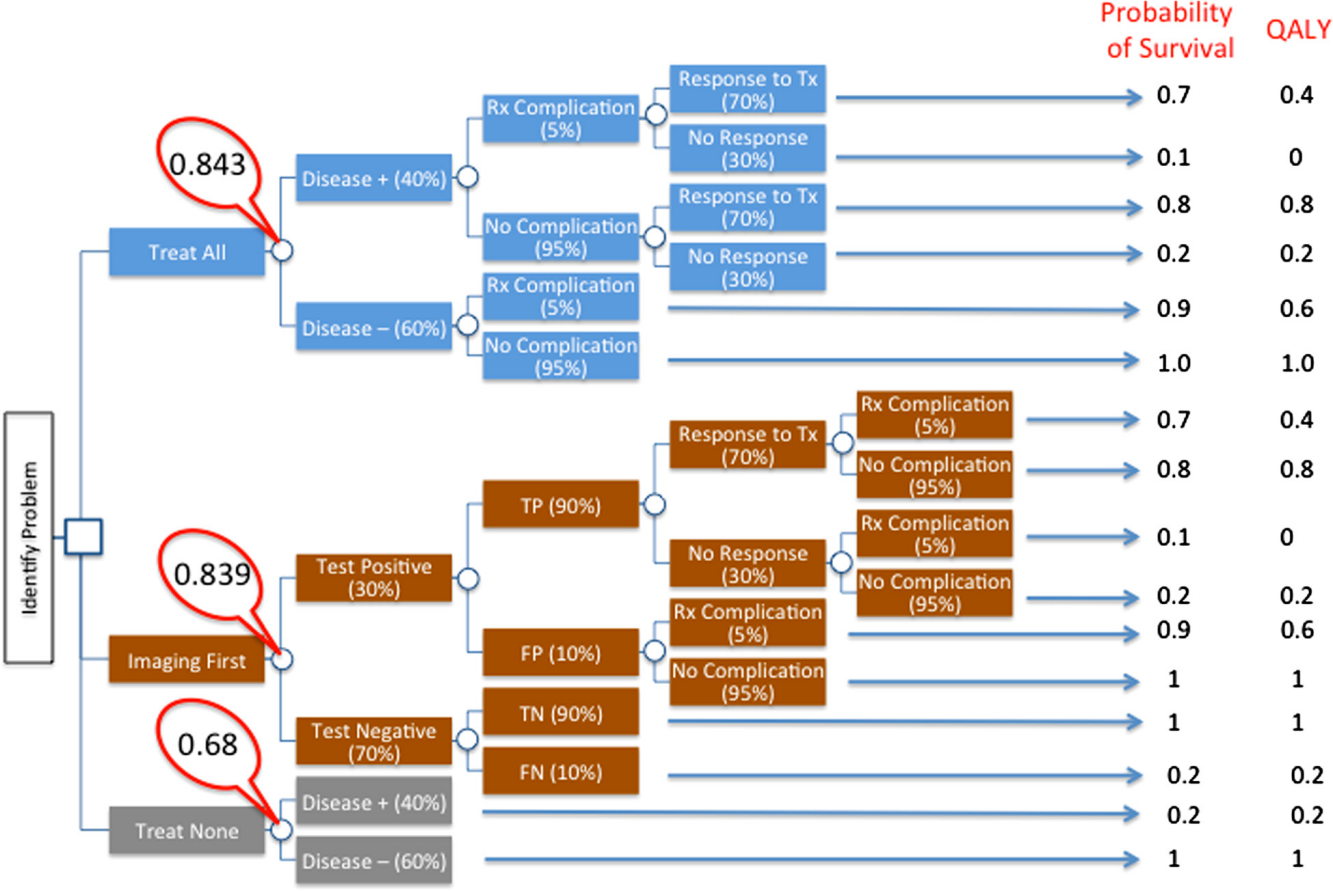
**A hypothetic decision analysis when 3 competing management options exist for a serious condition with a high disease prevalence of 40%.** Management options include a) treat all without testing, b) imaging test first and treatment guided by imaging findings, and c) treat none. The only available treatment is associated with a complication (5%) and only a fraction (70%) of all affected patients will respond to the treatment. The imaging test available has positive and negative predictive values of both 90%. Note that in this decision tree, the decision node is indicated by a square and chance nodes are indicated by circles. Probabilities of outcomes that branch from a chance node always add up to 1. In this example, when one only considers probability of survival, folding back and averaging out the decision tree will yield an average survival rate of 84.3%, which compares favorably to 83.9% of imaging first and 68% treatment none. However, when QALY was considered (after adjustment for the poor quality of life in patients who suffered from treatment complications), the treat all option only yielded an average of 82.9% which was inferior to the imaging first option with an average of 83.6%. Note that in this example, complication from the imaging test itself was not modelled.

Before a CEA analysis is performed, it is important to formulate the therapeutic and diagnostic options of the clinical questions at hand using steps of decision analysis. These steps in general include: 1) Identify and bound the clinical problem: this involves formulating the general diagnostic and therapeutic options that exist in the clinical question of interest (Figure 2 and Figure 3). Enter data for informed assessment: this includes entry of data such as probabilities of disease, complications, or outcomes. In addition, values are assigned to different possible outcomes. Any known and quantifiable average risk and success probabilities described in step 1 should be entered, 3) fold-back and average-out: this includes multiplying the probably of an event by the value assigned to the outcome. The multiplied products of all decision possibilities are then “folded-back” to the decision node where an average value can be obtained by summating all multiplied products downstream from the decision node, and 4) model uncertainties: this step involves sensitivity analysis where uncertainties that exist within the model can be adjusted in order to forecast the best mode of decision within the range of the uncertainties. There are commercially available software that allow building of decision trees and analysis of models applicable in CEA (e.g. Tree age, Syncopation).

### Comparing modalities in the “real-world” from observational registries

Imaging modalities often become incorporated in clinical diagnostic use within a short period of time after establishing technical feasibility and single-center diagnostic utilities, thus negating the opportunity to assess CEA of a new test before widespread clinical adaptation. On the other hand, large-scale randomized clinical trials comparing CEA of different imaging modalities are expensive, may not adequately represent the real-world practice settings, and may not keep pace with the rapid change in therapy available clinically. One method of assessing the incremental C/E ratio is by matching a patient cohort of a new modality, against a control group (usually from a large observational registry of patients) with similar test indications who had undergone an established clinical routine modality. A propensity score (between 0 and 1) for each patient in the cohort is then derived and then used to find the “best-match” amongst patients in the control group with similar baseline characteristics. This reduces the bias in patient characteristics and improves the comparability between the cohort and the control groups. Examples of 2 matching macro algorithms using SAS can be found in references [20,21].

### Determining costs

At the heart of a cost effective analysis is the accurate determination of cost and quantification of benefit or effect. Measuring or modelling both of these parameters can be complex. As we will discuss, this is only amplified when applying this paradigm to diagnostic cardiac imaging. The costs of a diagnostic imaging test include the fees associated with performing the test as well downstream costs associated with the imaging test. This could include changes in medical therapy, additional diagnostic tests, and therapeutic procedures. There are wide ranges of cost estimates for diagnostic imaging tests. In the United States, it can be difficult to accurately measure actual cost. In many instances, there is complex accounting with cost to charge ratios to reflect the difference between what is charged or billed and actual cost. The ‘charge’ is the price the consumer is billed for the service and can be highly variable depending on the health system. The actual cost is often a fraction of the charge and varies among hospitals, states, and third party payers. Consequently, many investigators use cost estimates typically derived from the United States’ Centers for Medicare and Medicaid Services (CMS) average payments. These cost estimates published by the CMS can be looked up online using the Healthcare Common Procedure Coding System (HCPCS) or Current Procedural Technology (CPT) codes of the procedures (http://www.cms.gov/apps/physician-fee-schedule/).

The cost of an imaging test includes the cost of using and maintaining the equipment (technical fee) and the cost of the interpretation (professional fee). The costs of the medications used in stress imaging and the contrast agents also factor into the costs. There is great variability in the price (or charge) of CMR within the U.S. The 2012 Medicare global national average for a stress MRI study with contrast was $672.24 [22]. However, there is substantial variability in price among hospitals, insurance providers, and to the extent of what payers will reimburse. In contrast, the estimated price of CMR across the globe appeared to be lower: Germany (164–393 Euros), United Kingdom (558 Pounds), and Switzerland (1420 Francs) [23]. Even within the context of a clinical trial, the NIH research rate for CMR is a fraction of the typical clinical charge at most institutions. This inherent variability in cost can be a barrier for accurate CEA, as the cost data at best is an approximation of costs from a societal perspective. At worst, the cost represents an artificial estimate with little bearing on real world economics. Since the purpose of a CEA in CMR is to link an imaging test to a therapeutic treatment plan/strategy and then ultimately to an outcome, all of the downstream costs associated with the imaging test are also included. This includes the cost of medications, additional diagnostic tests, and therapeutic procedures. It can also include indirect and intangible costs of the resulting health effect. As discussed above, there is presently no universal or precise method of evaluating the costs of tangible health care interventions, let alone health care decisions with a multitude of opportunity costs. Assessment of direct costs (e.g., physician time, supplies, etc.) varies across different institutions, regions, and nations, in part due to variability in billing and insurance remuneration. This further complicates the accuracy and generalizability of CEA.

### Determining effectiveness

Measuring the effectiveness of a diagnostic imaging test in a clinical setting is complex for multiple reasons. The majority of clinical trials evaluating the performance of an imaging test have examined diagnostic accuracy. Linking an imaging test, subsequent therapy, and outcome can be difficult even within the context of a carefully conducted trial, owing to individual patient and provider behaviors [24]. Imaging tests can only capture a snapshot of cardiac structure or physiology, and provide an estimation of the natural progression of the disease of interest. In addition, linking an imaging test or a therapy to an improvement in survival is difficult; accordingly, improvements in quality of life predominate, including reductions in “hard” clinical end-points such as myocardial infarction, coronary revascularization, hospitalizations for congestive heart failure. From a patient perspective including patient satisfaction and improvements in functional capacity are relevant and may be included in CEA of imaging tests.

### Applying results of CEA

The utilitarian application of CEA to medical decision-making may be problematic and overly simplistic in a number of clinical scenarios. Providing effective healthcare often involves liberal, ethical considerations, which in certain cases may include the provision of therapy that is poorly cost-effective [15]. For example, the provision of recombinant enzyme therapies for rare genetic disorders is expensive, but is offered in some health care systems for ethical reasons. In the current health care climate of the United States, physicians and payers will seldom limit or withhold a potential intervention on the basis of cost effectiveness. In addition, thresholds for what is considered cost-effective may also differ based on the implications of the disease, therapy, and relative wealth of the health care economy in question [19]. These caveats further limit both conducting and applying CEA to guide medical decision-making.

Cost effectiveness can be a useful tool to guide decisions on resource-allocation, but it is equally important to understand that it only represents a limited dimension of decision-making. Individual preferences, cultural factors, political pressure, affordability, and other constraints such as human resources and equipment often could not be considered in CEA.

### Challenges in conducting CEA in cardiac imaging

There are a number of challenges in performing a CEA for cardiac imaging. Historically, the performance of an imaging test has been evaluated in terms of diagnostic accuracy and prognostic utility. In this regard, performance characteristics such as sensitivity, specificity, positive and negative predictive value serve as the commonly used metrics [25,26]. The downstream decision-making and resource utilization that occurs after a diagnostic test is complex and involves a number of factors (including patient preference and heterogeneous clinical practice patterns) that are difficult to model.

Economic analyses primarily evaluating medical interventions with specific outcome measures are more conducive to CEA, as compared with diagnostic imaging, which may only incrementally improve the ability to formulate a diagnosis, or serve as a component in a clinical evaluation algorithm to guide therapeutic decisions. Therefore, there are multiple facets to consider for a cardiac imaging CEA. First, there must be some meaningful threshold for a diagnostic result. This may be problematic, as potentially subjective aspects of imaging assessment may not be conducive to reproducible and widely acceptable thresholds. Furthermore, imaging results may have multiple components that are internally inconsistent. Second, and most importantly, the result of the incremental value of the imaging study must impact clinical outcomes for the patients evaluated. This latter component requires not only consideration of the diagnostic imaging study, but also the cost-effectiveness of any subsequent therapies instituted as a result of imaging tests. This complicates trial design, as investigators must also account for the efficacy and cost of potential therapies instituted by diagnostic test results, as well as ensuring strict protocol adherence to management strategies with minimization of patient crossover. Additionally, it should be emphasized that the main question being evaluated in a CEA is the incremental value of the diagnostic imaging study beyond existing, or standard clinical assessment. This point is often poorly addressed in diagnostic imaging CEA studies that may focus on diagnostic accuracy, as opposed to independent, incremental effect on hard clinical endpoints.

A comprehensive CEA of a diagnostic modality requires the assessment of a number of parameters, including accuracy of testing, efficacy of subsequent imaging guided therapy, economic and health costs of imaging (example: ionizing radiation from nuclear perfusion imaging), health and economic benefits of imaging-guided decisions, and costs from inaccurate results or repeat testing. This complexity increases the probability of bias and inaccuracy of results. Therefore, clinical trials with larger sample sizes are required, which are often cost prohibitive.

An important aspect of CEA design relates to the prevalence and severity of disease in the population being evaluated [27]. According to Bayesian theorem, the pre-test prevalence of disease has substantial impact on the diagnostic performance of the particular test being evaluated. Furthermore, the efficacy of downstream therapies guided by imaging results may be very different based on the population being treated [28]. Therefore, validity of a particular CEA in one patient population is not necessarily generalizable to other populations; for example, CEA of CMR in symptomatic chest pain patients cannot be applied to asymptomatic patients at risk for CAD. This requires CEA studies in differing populations based on the pre-test prevalence and severity of the particular disease being evaluated, and leads to inflation of costs related to conducting CEA for any particular diagnostic imaging modality. Finally, in clinical scenarios evaluating diagnostic imaging modalities as a screening tool, the relatively lower prevalence of cardiac disease within these populations requires large sample sizes, followed for longer periods of time, which may also be cost prohibitive.

The need for larger studies to evaluate cost-effectiveness creates another obstacle related to the extent that local experience and expertise may alter precision of study outcomes. Larger samples sizes often require multiple sites, which may have varying expertise with different cardiac imaging modalities. One example includes echocardiographic assessment of dyssynchrony for cardiac resynchronization therapy. Despite numerous early single center studies demonstrating promising results, a larger, multicenter trial found no benefit for echocardiographic indices of dyssynchrony, and more importantly demonstrated a high variance of imaging measurements between different centers [29]. This study highlights the challenge of performing large multicenter CEA studies that ensure both accurate and precise assessment of the diagnostic technologies in question.

Physician adherence to CEA study design protocols may also be difficult to ensure. Physicians may utilize non-evidence based clinical information to guide decision-making. Shaw et al. reported significant variance in clinical decision-making after presentation of nuclear perfusion imaging results of symptomatic chest pain patients to clinicians [30]. This variability in practice further complicates trial design that requires adherence to downstream management protocols based on imaging results. Another significant obstacle to CEA is the pace of advancement of cardiac imaging. Within the past decade, substantial technological improvements have been made in many novel cardiac imaging techniques (CMR, CCTA) that have improved diagnostic yield, and the cost of performing these diagnostic studies. This rapid evolution of the technology can quickly outdate CEA studies performed with earlier iterations of imaging techniques.

### CMR and CEA

Current ongoing trials addressing cost effectiveness in imaging remain limited especially on comparing the cost-effectiveness of different modalities. In this context, it remains unclear that stress CMR represents an important advance beyond other stress imaging modalities in reducing health care costs or improving effectiveness of patient care. While CMR is considered to be a costly technology, it has established several lines of evidence that potentially position it to be a viable contestant in competing with other imaging tests in a CEA. First, CMR is more accurate in detecting coronary artery disease when compared to other imaging modalities. In a head-to-head comparison with dobutamine-stress echocardiography, dobutamine stress CMR exhibited higher diagnostic sensitivity (86% vs. 74%) as well as specificity (86% to 70%) [31]. Multicenter evidence from the MR-IMPACT 2 trial demonstrated superior sensitivity but inferior specificity of stress perfusion CMR compared to SPECT in the context of suspected CAD [32]. Another recent prospective single center trial, CE-MARC also reported that the sensitivity of CMR was found to be superior to SPECT (86.5% vs. 66.5%) while the specificity was similar (83.4% vs. 82.6%) [33].

Two recent publications have applied a decision analytic model using data from CE-MARC to evaluate the cost-effectiveness of CMR in patients with suspected coronary artery disease. Walker et al. compared 8 different testing strategies involving ETT, SPECT, CMR, and coronary angiography in a United Kingdom National Health Service model. Using the threshold of £20 K – 30 K per QALY only two strategies were cost-effective: 1) CMR after a positive or inconclusive ETT, followed by coronary angiography if CMR was positive or inconclusive, 2) CMR as a first line test followed by coronary angiography if CMR positive or inconclusive [34]. In this analysis health related outcomes were estimated using a previously published Markovian model to predict future cardiac events and mortality in patients with coronary artery disease. In another study, Boldt et al. used the diagnostic performance of SPECT and CMR from the CE-MARC trial to develop a model based on Bayes’ theorem comparing the cost-effectiveness of CMR with SPECT for the diagnosis of CAD in a German economic system [35]. This study focused on the perspectives of the health care payers, instead of society. In this model, CMR, despite being approximately 40% more expensive than SPECT, was found to be more cost-effective than SPECT in low to intermediate-risk patients when pre-test CAD probability was below 60%. Boldt et al. further commented that their results indicated invasive coronary angiography was more cost-effective than either noninvasive tests when pre-test CAD probability was > 60%, consistent with recent guideline recommendations. Both of these analyses are informative and suggest that despite a higher cost per study of CMR than SPECT, the higher diagnostic performance of CMR translates to improved outcomes downstream and cost-effectiveness. However, it is important to understand that these are analytic models and as such make several important assumptions which do not necessarily mirror real-world clinical practice. For example, in these models an abnormal or indeterminate stress test automatically results in a coronary angiogram which does not reflect current practice. In the analytic model by Walker et al. a significant stenosis by coronary angiography results in revascularization and thereby an estimated reduction in angina compared with medical therapy alone translating to an improvement in QALY. Fractional flow reserve (FFR) during cardiac catheterization, cardiac CT angiography, and the relative inexpensive stress echocardiography, which are either increasingly or commonly used in the diagnosis and management of coronary artery disease, were not included in these models. Since the CE-MARC trial was the primary source of diagnostic accuracies of CMR and SPECT in both studies, whether the results of these 2 CEA will be altered by different imaging equipment, local expertise, and reimbursement schemes is unclear. They however, highlight the important finding that stress CMR, given its high diagnostic accuracy, can be a cost-effective tool compared to SPECT. In addition, they underscore the significant need for prospective comparative effectiveness trials comparing different imaging strategies for the management of coronary artery disease.

To date, several single center studies investigating the cost of CMR in adjudicating risk have been applied to two other major referral populations: patients with acute chest pain and new-onset cardiomyopathy. Assomull et al. reported that the performance characteristics of a contrast-enhanced CMR (consisting of coronary angiography and late gadolinium enhancement) were equivalent to an initial strategy of invasive coronary angiography to adjudicate the etiology of heart failure in 120 consecutive patients with new-onset systolic dysfunction. In addition, using a decision analytic framework characteristic to CEA analyses, these authors further demonstrated that (relative to costs in the United Kingdom), an initial approach using CMR as a “gatekeeper” to provisional invasive angiography was cheaper than invasive angiography in all patients (net 26% cost savings for CMR-guided approach) [36]. It is important to note that this was a cost minimization analysis given that all patients received both cardiac catheterization and CMR and thus cannot conclude that CMR was more effective. Registry data from the Euro-CMR which evaluated over 11,000 patients, reported that CMR significantly impacted nearly two-thirds of the patients according to pre-specified definitions of “significant change in management,” and this included an important 16% minority of patients in whom an entirely new diagnosis was uncovered [37]. A substantial portion of this impact on patient management was related to the diagnosis and management of heart failure patients. These results collectively suggest that the use of CMR as an initial test in patients with heart failure may not only be diagnostically effective, but also yield significant cost savings.

CMR has also been investigated in the risk stratification of patients with acute chest pain. Miller and colleagues extended the concept of an emergency ward observation unit to include CMR imaging as pre-discharge risk stratification compared to standard of care which included inpatient admission. In this study of 109 patients with acute chest pain, there was no significant difference in clinical event rates between patients managed in the observation unit with CMR versus inpatient admission [38]. However, the cost of management in an inpatient unit was significantly higher than an observation unit with CMR ($4,742 vs. $3,101). No cases of acute coronary syndrome were missed by the CMR arm [39] a finding which was also reported by other studies [40] in a similar setting.

Evaluation of suspected coronary artery disease in the outpatient setting is the largest group of patients around which the increasing costs of imaging have become relevant. There remains limited data with regard to the cost effectiveness of CMR in this cohort. In a small study of 218 patients in Europe, CMR reduced the subsequent performance of angiography by nearly 63%, with a significant per-patient cost savings (greatest for those patients at lowest risk for CAD, but present across all risk strata) [40]. Both of these cost-minimization studies provided the foundation for larger, completed and ongoing studies (e.g., CE-MARC, ISCHEMIA), in which cost effectiveness of stress CMR for the assessment of coronary disease form a prominent role.

The high diagnostic capability of CMR owing to its technical advantages as well as the ability of a comprehensive CMR study to provide multiple cardiac structural and physiologic assessments can potentially reduce the downstream need for further testing, although this hypothesis remains to be tested. Many clinical studies have utilized this technical strength of CMR with the potential of reducing the need to perform multiple imaging studies for addressing multiple cardiac issues of interest (e.g. echocardiography for cardiac size and function, nuclear scintigraphy for myocardial perfusion). It is the opinion of the authors that, with technical strengths mentioned above, CMR may be well positioned to improve clinical effectiveness in the planning and performance of a growing number of invasive procedures when performed in specific patient subsets and indications (e.g. cardiac resynchronization therapy, ICD implantation, radiofrequency ablation of arrhythmia). Early evidence of this potential benefit already exists [41-43].

### CEA for CMR: a global perspective

**Figure 4:**
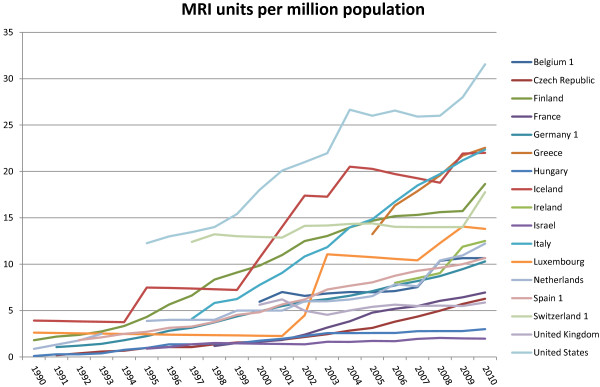
**MRI units per one million populations across different European nations.** Source: OECD [48].

European researchers have not only succeeded in pushing forward the technical boundaries in CMR, but have been prominent in contributing to the literature supporting the application of the technique in real world clinical practice [33,37,44]. Preliminary cost effectiveness analyses have been performed in the evaluation of patients with stable coronary artery disease [45,46]. The highly anticipated EVINCI study which concluded data collection in June 2012, should provide important data in establishing the cost effectiveness of various imaging modalities in the evaluation of symptomatic patients with suspected coronary artery disease [47]. There are considerable differences among European nations with respect to cardiovascular disease prevalence, morbidity, and mortality. Likewise there are differences in practice patterns and resources amongst the different nations [45,46]. Comparisons between aggregate data on the numbers of medical personnel, nurses and pharmacists per million such as that published in the “Health at a Glance” database produced by the Organization for Economic Cooperation and Development (OECD) reveal substantial differences between European nations. Total expenditure per annum on health varies more than 100 fold, from $46 dollars per capita in Moldova to $5900 in Luxemburg [48]. Similarly there are differences in the number of MRI units per million population (Figure 4) which will impact utilization and as a result local practice patterns.

The significant differences between the health systems of Europe described above pose important challenges in conjunction with the lack of evidence or consensus as to the optimal methods of considering cost and economic issues [49] and make the evaluation of the cost effectiveness of CMR in Europe as a whole a very complex challenge. It is likely that there will be variability in cost utility analysis from region to region, but collaborative efforts between national societies and the European Society of Cardiology though its working group on CMR to facilitate, promote and transmit high quality research in this area has the potential to improve the application of this technology in the future.

As in North America and Europe, the use of CMR in many Asian nations has also increased. Within China, Fuwai Hospital performed approximately 12,000 CMR studies between the years for 2004–2010 [50]. Similar growth is expected for nations such as Japan, India, South Korea and Thailand though comprehensive national statistics have not been published. Given differences in health care systems, disease prevalence, socioeconomic heterogeneity, and culture differences the Asian Society of Cardiovascular Imaging released appropriateness criteria for the use of CMR in 2010 [51]. In contrast to the United States which has seen a relative reduction in government funding for medical research, nations like China, India, South Korea, Taiwan, and Singapore have increased national funding for scientific research [52] setting the stage for further clinical research in cardiac imaging and CMR in particular. Given the significant heterogeneity in economic systems and health care practice, cost-effectiveness studies will also be important to define the role of CMR within Asian countries.

## Conclusion

CMR has experienced tremendous growth over the last decade with data supporting its excellent diagnostic and prognostic performance in conditions such as ischemic heart disease, cardiomyopathies, and congenital heart disease. Though CMR accounts for only a small fraction of cardiac imaging expenditures within the U.S., demonstrating its cost-effectiveness across a spectrum of disease will be critical to ensure continued growth and appropriate utilization. Cost-effective analyses will have to draw on large randomized, controlled studies using a CMR guided treatment strategy as well as registry data to reflect real world clinical practice. As the health care environment continues to evolve it is clear that fiscal constraints on cardiac imaging will be necessary as the current rate of growth is unsustainable in many parts of the world. Cost-effectiveness analysis will be pivotal in demonstrating the value of CMR and its impact on patient outcomes.

## Abbreviations

CMR: Cardiovascular magnetic resonance; CEA: Cost-effectiveness analysis; QALY: Quality adjusted life year; CMS: Centers for Medicare and Medicaid Services; CCTA: Cardiac computed tomography angiography; SPECT: Single photon emission computed tomography.

## Competing interests

The authors declare that they have no competing interests.

## Authors’ contributions

All authors (SF, CD, BH, SA, RS, RK) have made substantial contributions to the conception, design, drafting, and critical revision of the manuscript. All authors read and approved the final manuscript.
